# Impacts of multivalued interventions on maize farmers’ welfare: Evidence from SIPMA development project in Ghana

**DOI:** 10.1016/j.heliyon.2024.e40325

**Published:** 2024-11-13

**Authors:** Bright O. Asante, Stephen Prah, Omphile Temoso, Forster Boateng, Abubakar Gyinadu

**Affiliations:** a Department of Agricultural Economics, Agribusiness and Extension, Kwame Nkrumah University of Science and Technology, Kumasi, Ghana; b Department of Agribusiness, Applied Economics and Agriscience Education, North Carolina A&T State University, Greensboro, NC, USA; c UNE Business School, University of New England, Armidale, NSW, 2351, Australia; d Tree Crops Development Authority (TCDA), M9F9+9X3, Lesley Opoku-Ware Drive, Kumasi, Ghana; e Department of Agricultural Economics and Agribusiness, University of Ghana Legon, Ghana

**Keywords:** Agriculture, Interventions, Welfare, Development, Multivalued IPWRA, Ghana

## Abstract

Recent research has highlighted the significance of agricultural development programmes in Sub-Saharan Africa for enhancing production, food security, and farmer welfare. However, these studies frequently investigate the effects of a single intervention rather than a combination of interventions. This study examines the impact of three interventions (credit access, structured market, and entrepreneurial training) on maize yield, food security, food expenditure and non-food expenditure in Ghana. We utilized multivalued inverse probability weighted regression correction, and propensity score matching with data from the Smallholder Inclusive Productivity and Market Access (SIPMA) agricultural development programme on 477 maize farmers. Participation in SIPMA interventions significantly increased maize yields, farm income, food spending, and non-food spending. For smallholder farmers, participation in a combination of input credit provision, structured market, and entrepreneurial training results in the highest yield, farm income, and non-food expenditures, while participation in entrepreneurial training alone results in a lower yield. Our empirical evidence suggests that the interventions should be expanded to other communities because the project's characteristics are distinctive in terms of fostering community engagement and collaboration with local/regional institutions.

## 1 Introduction

According to the United Nations Conference on Trade and Development [1], 119 million people in 26 least-developed countries and 800 million people worldwide are food insecure due to increased food prices and trade restrictions caused by the COVID-19 pandemic and Russian-Ukraine conflict. To address these concerns, agricultural development projects and programmes have been implemented throughout Africa, including in Ghana, with the goal of improving agricultural productivity, crop yields, farm revenue, and welfare for farming households and other value chain actors [2,3].

Agriculture plays a crucial role in Ghana’s economic growth, particularly in rural areas where it supports the livelihoods of a large portion of the population. Maize is a key staple food and income source for smallholder farmers. However, challenges such as limited access to input credit, underdeveloped market structures, and insufficient entrepreneurial skills hinder farmers from achieving optimal yields, increasing farm income, and enhancing their economic well-being. Input credit is vital for smallholder maize farmers, enabling them to acquire necessary inputs like seeds, fertilizers, and pesticides, which are essential for improving crop yields and harvest quality. Unfortunately, access to input credit is often constrained by high interest rates, lack of collateral, and perceived risks in lending to small-scale farmers ([4]; Dzanku & Sarpong, 2020). These barriers lead to suboptimal input use, resulting in lower yields and reduced farm income [[4], [5], [6]].

Structured markets provide a fair and reliable platform for farmers to sell their produce, offering stability in pricing and demand, which is crucial for encouraging investment in productivity-enhancing practices [4,7,8]. In Ghana, the absence of well-organized markets often results in low prices and unpredictable demand, undermining farm income and discouraging investment [[9], [10], [11]]. Entrepreneurship training is essential for equipping farmers with the skills needed to manage their farms as businesses, optimize resource use, and adapt to market changes [10,12]. Such training supports improved farm management, market access, and income diversification, contributing to sustainable growth and economic resilience [[9], [10], [11]]. The synergy of access to input loans, structured markets, and entrepreneurial training is critical for improving maize farmers’ livelihoods in Ghana. It encourages investment in quality inputs, provides consistent sales revenue, and empowers strategic decision-making, resulting in improved productivity, increased income and improved welfare of maize farmers in Ghana.

Several studies have been undertaken to assess the impact of agricultural development interventions on a variety of economic outcomes and geographies ([13,14]; Biru et al., 2020; [12,[15], [16], [17], [18], [19]]; Bizikova et al., 2020; [20,21]). Some studies found these interventions to improve diets and income (Bizikova et al., 2020; [16]), food security [2], and household well-being (Biru et al., 2020; [6,13,[22], [23], [24], [25]]). Other studies found such interventions to reduce productivity gaps and set farmers on a new path towards production technological innovation [19,[26], [27], [28]].

However, the findings of previous investigations remain inconclusive. Satapathy et al. [29], for example, found that participation in development projects has a beneficial effect on consumer behavior, income transfers, and educational achievement. Iddrisu et al. [30] found participation in the voluntary cocoa certification process to enhance smallholder income and yield levels. In Kenya, Nechifor et al. [31] found participation to have a positive effect on calorie intake. Karlan & Zinman [32] found that the expansion of credit supply has a positive impact on overall welfare. They further indicated that the individuals who received the expanded credit access experienced tangible benefits. Karlan and Valdivia [33] used randomized control trial and found that additional entrepreneurship training showed a positive impact on key outcomes such as business revenue, profits, and employment. On the other hand, some studies found participation in food security programmes to have a negative impact on household food security [30], while others report only a marginal effect on smallholder farmers' welfare (e.g., [17,18]; Gebre et al., 2021). Thus, more research is required to determine the true impact of access to credit, structured markets, and entrepreneurial training on crop yield and food security.

Furthermore, most impact studies have been skewed towards addressing solely output risk (productivity), with few addressing both production and price risks (resilience). Given that price is a fundamental predictor of welfare outcomes such as income and consumer expenditure, it is surprising that only a few studies have addressed both price and output risks. Additionally, while the factors influencing farmers' participation in productivity improvement programmes are well documented in the literature, there is a lack of information on potential factors influencing smallholders' decision to participate in development projects.

Thus, the purpose of this study is to contribute to the existing empirical literature on agricultural development projects by examining the effect of participation in three development interventions (access to input credit, structured markets, and entrepreneurship trainings) delivered through the Smallholder Inclusive Productivity and Market Access (SIPMA) project on the welfare of smallholder farmers in Ghana. The SIPMA programme is a food security intervention designed by the SIPMA Consortium and implemented by the Alliance for a Green Revolution in Africa (AGRA) [34], with the objective of improving productivity through enhanced market access through these interventions. The project assisted approximately 143,000 smallholder farmers in Ghana with farm inputs (such as seed and fertilizer), training on good agricultural practices, access to structured markets, credit provision, agricultural mechanizations, and enterprise development. The SIPMA Project differs from other food security initiatives in that it targeted pricing and production issues in agriculture.

Unusually for impact studies, we are investigating the effects of a combination of interventions in addition to a single intervention. In our analysis, a farmer has eight mutually exclusive options for SIPMA interventions he/she could participate in: *nonparticipation only*, *input credit provision only*, *structured market only*, *entrepreneurial training only*, *combination of input credit provision and structured market*, *combination of structured market and entrepreneurial training*, and *combination of input credit provision, structured market, and entrepreneurial training*. To maximize their utility, farmers select one of eight possible combinations. The findings of this study have implications for development researchers and practitioners because they can help answer questions such as whether participation in multiple development interventions provides smallholder farmers with greater benefits than participation in a single intervention. Which combinations of development interventions appear to have the greatest effect on farmer yield, food security, food and non-food expenditure?

To achieve our objectives, we used three robust methods: inverse probability weighted regression adjustment (IPWRA), propensity score matching (PSM), and multivalued inverse probability weighted regression adjustment (MIPWRA). As noted by Olagunju et al. [13] and Ton et al. [14], few studies that evaluate the impact of agricultural development projects use robust methods. They are restricted to those utilizing conditional instrumental variable treatment effects [13], the SWAT model [19,35], and the correlated random effect ordered probit estimator [13,20]. However, we are unaware of any studies that have evaluated the impact of participation in programmes that provide access to credit, structured markets, and entrepreneurship training while considering the possibility of endogeneity.

This study estimates the factors influencing participation in access to credit, structured market, and entrepreneurship training, as well as their impact on welfare, utilizing IPWRA, MIPWRA, and PSM. Since participation in access to credit, structured markets, and entrepreneurial training are not randomly assigned to Ghanaian farmers, the ATET estimates were utilized to account for selection bias. To account for the endogeneity issue that arises in empirical studies when the exogenous variables examined in the model have a correlation with the error term, a multivalued inverse probability-weighted regression adjustment was implemented. In our case, endogeneity may be an issue because participation, for instance, does not always result in increased yield, food security, and well-being for beneficiaries.

The next section of this paper presents the methodology including the study area, data and sampling procedure, and empirical strategy. The results and discussion are the next section after the methodology whereas conclusions and policy recommendations conclude the paper.

## 2 Methodology

### 2.1 Study area

**Fig. 1:**
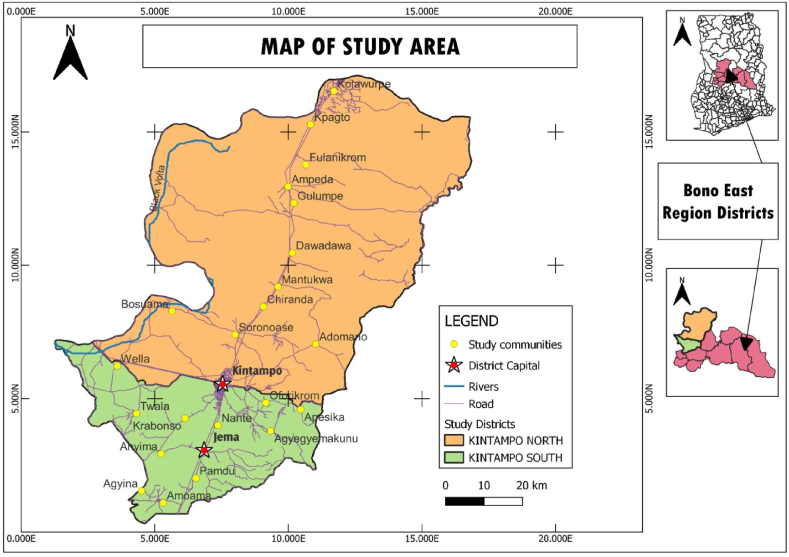
Map shows the selected communities, and districts in the Brong-Ahafo region.

Source: Authors’ design, 2021.

The study was conducted in the Brong-Ahafo region of Ghana. The region is one of the second largest region in Ghana located within longitudes 0^0^ 15’ E−3^0^ W and 8^0^ 45’ N-7^0^ 30’ S in the southern part of Ghana. It covers land area of 39,558 km^2^ and bordered with Northern region (North); Ashanti and Western regions (South); Volta region (East); and Eastern region (South-east). Fig. 1 shows the map of the study area. It has a population of approximately 2,282,128 people, 69.1 % of whom rely on agriculture, and 70 % of whom depend on crop production (GSS, 2021). The region has fertile soil, favorable climatic conditions, tourist attractions, and a diverse cover of vegetation. In addition, it is regarded as an agriculturally based economic activity that contributes approximately 30 % of Ghana's domestic food supply (GSS, 2021). Among the crops grown are maize, rice, yam, cassava, sorghum, cashews, and cacao, among others. Maize produced approximately 14,111 Mt more than other food crops in the region, according to the GSS (2021).

### 2.2 Data and sampling procedure

Our target population was maize farmers in Ghana, specifically Brong-Ahafo region. Both primary data and secondary information were utilized in this study. Primary data was obtained through a structured questionnaire on farmers’ socioeconomic characteristics, institutional factors, and production variables. The data collection was conducted by trained enumerators between July and August 2021. The secondary information was obtained from the Ministry of Food and Agriculture (MoFA) website, Food and Agriculture Organization website, and journals.

To determine the sample size, we employed the Yamane’s formula which was specified in equations (1), (2), (3) as:

(1) $$n=\frac{N}{1+N{\left(e\right)}^{2}}$$

where n = sample size, N = targeted maize farmers population (31,073), and margin of error (5 %).

(2) $$n=\frac{31073}{1+31073{\left(0.05\right)}^{2}}$$

(3) $$n=\frac{31073}{1+77.6825}$$

n=395

Farmers were sampled using a multistage sampling technique and a structured questionnaire. First, we selected the Brong-Ahafo Region of Ghana on purpose due to the prevalence of SIPMA interventions and the region's high maize production. Also, we sampled purposively the Kintampo North and South districts for the same reasons. Each district's twelve (12) communities were randomly divided into six (6) beneficiary communities and six (6) control communities. A list of maize farmers was obtained from the local agricultural extension offices under the Ministry of Food and Agriculture. Twenty-five (25) farmers were selected at random from each community and interviewed. We over sampled and collected information from 477 farmers, including 212 beneficiaries and 265 non-beneficiaries, for an effective high response rate. Prior to the final data collection, a pilot survey was conducted to ensure that all modifications to the questionnaire were incorporated.

### 2.3 Econometric framework

To estimate the impact of participation in agricultural intervention on smallholder farmers’ welfare, the average treatment on treated (ATT) was used. The average difference in outcomes between participants and non-participants is called the ATT (Takahashi & Barrett, 2013). The ATT is specified in equation (4) as:

(4) $$ATT=E\left\{Z_{iP}-Z_{iNP}|W_{i}=1\right\},=E\left(Z_{iP}|W_{i}=1\right)-E\left(Z_{iNP}|W_{i}=1\right)$$

where; $Z_{iP}$ and $Z_{iNP}$ represents potential outcome for participant and non-participants, respectively; $W_{i}$ represents the treatment status thus 1 = participant and 0 = otherwise; E{.} represents expected operator. From the above equation, it obvious that we cannot observe the outcome of participants had they not participated given as $E\left(Z_{iNP}|W_{i}=1\right)$. Nevertheless, the unobserved counterfactuals are replaced by the outcomes of non-participants given as $E\left(Z_{iNP}|W_{i}=0\right)$. Therefore, this replacement may lead to biased ATT results (Takahashi & Barrett, 2013; [36]).

To best address the above problem, Wooldridge (2010) opined the inverse probability weighted regression adjustment (IPWRA). The inverse probability weighted regression adjustment (IPWRA) also used as robustness check for missing data problem to calculate each predicted outcomes averages of the treatment. For double robust characteristics, we were able to achieve correctly and consistently specified estimates (Cattaneo, 2010).

We first computed the inverse probability weights (IPW) by weighing the observations from the inverse probability of being participated. The propensity scores based on the Rosenbaum and Rubin (1983) approach are specified as:

(5) $$p\left(X\right)=\mathrm{Pr}\left(W_{i}=1|X\right)=G\left\{h\left(X\right)\right\}=E\left(W_{i}|X\right)$$

where; X and G{.} represents vector of observed factors of pre-treatment covariates and cumulative distribution function, respectively. Table 1 presents the definition of selected variables such as socioeconomic and institutional factors used in the model. Further, a synthetic sample was then created by generating the propensity scores (equation (5)) to distinguish the covariates from the assignment of treatment. According to Hirano and Imbens (2001), the weights computed by adopting inverse weights equal to 1 for participant and $\frac{\hat{p}\left(X\right)}{\left(1-\hat{p}\left(X\right)\right)}$ for non-participant are combined in equation (6) as:

(6) $$wt_{i}=W_{i}+\left(1-W_{i}\right)\frac{\hat{p}\left(X\right)}{(1-\hat{p}\left(X\right)}$$

where; $wt_{i}$ and $\hat{p}$ represents the weights and estimated propensity scores, respectively.

**Table 1:** Explanatory variables used in the models.

Variables	Measurement	Expected sign
Age	Years	+/−
Sex	1 if a farmer is male and 0 = otherwise	+/−
Residential status	1 if a farmer is indigene and 0 = settler	+/−
Education	Years	+
Off-farm activity	1 if a farmer engaged in off farm activity and 0 = otherwise	+/−
Maize experience	Years	+
Household size	Number	+
Plots	Number	+
Farm size	Acre	
Land ownership	1 if a farmer owned land and 0 = otherwise	+
Credit	1 if a farmer had access to credit and 0 = otherwise	+
Structured markets	1 if a farmer had access structured market and 0 = otherwise	+
Extension contacts	1 if a farmer had extension contact and 0 = otherwise	+
Extension distance	Distance from farmer’s homestead to the nearest extension office in kilometers	
Interest free credit	1 if a farmer had interest free credit and 0 = otherwise	+
FBO membership	1 if a farmer is an FBO member and 0 = otherwise	
Market distance	Distance from farmer’s homestead to the nearest market in kilometers	

In addition to the IPW, the regression adjustment (RA) was estimated because it gives a linear regression model for participant and non-participant and averages of the predicted welfare indicators (yield, farm income, food expenditure and non-food expenditure) to compute the treatment effects. A clear difference between the RA and IPW is that the former provides the outcome estimates and the latter focus on treatment effect computations. We compute the ATT for RA based on Wooldridge (2010) expressed in equation (7) as:

(7) $$ATT_{RA}=n_{P}^{-1}\sum_{i=1}^{n}W_{i}\left[r_{P}\left(X,\lambda_{P}\right)-r_{NP}\left(X,\lambda_{NP}\right)\right]$$

where; $n_{P}$ represent number of participants, $r_{i}\left(X\right)$ represent participants and non-participants regression model based on observed covariates X and coefficients $\lambda_{i}=\left(\sigma_{i},\beta_{i}\right)$.

Further, we computed the IPWRA estimator by combining inverse probability weights and regression adjustment. According to Wooldridge (2010), correctly specifying of either IPW or RA provides more consistent estimates of the treatment effects conditioned on the covariates given. In order words, whether one of the models is specified correctly or not, the results of treatment effect are still consistent. The IPWRA estimator for ATT is specified as:

(8) $$ATT_{IPWRA}=n_{P}^{-1}\sum_{i=1}^{n}W_{i}\left[r_{P}^{*}\left(X,\lambda_{P}^{*}\right)-r_{NP}^{*}\left(X,\lambda_{NP}^{*}\right)\right]$$

where; $\lambda_{P}^{*}=\left(\sigma_{P}^{*},\beta_{P}^{*}\right)$ is generated from the weighted regression

(9) $$\min_{\sigma_{P}^{*},\beta_{P}^{*}}\sum_{i=i}^{N}W_{i}{\left(z_{i}-\sigma_{P}^{*}-X\beta_{P}^{*}\right)}^{2}/\hat{p}\left(X,\hat{\psi }\right)$$

and $\lambda_{NP}^{*}=\left(\sigma_{NP}^{*},\beta_{NP}^{*}\right)$ is generated from the weighted regression

(10) $$\min_{\sigma_{NP}^{*},\beta_{NP}^{*}}\sum_{i=i}^{N}\left(1-W_{i}\right){\left(z_{i}-\sigma_{NP}^{*}-X\beta_{NP}^{*}\right)}^{2}/\left(1-\hat{p}\left(X,\hat{\psi }\right)\right)$$

From the above equations (8), (9), (10), we compared the ATT for RA and ATT for IPWRA as both have common expression, however, different weighted estimates were based on the regression coefficients (Wooldridge 2010).

Generally, the IPWRA model depends on two assumptions with the first assumption being the conditional independence assumption (CIA). The CIA postulated that treatment assignment is mostly randomized when condition on a set of covariates. Self-selection into treatment assignment is a strong and controversial assumption due to unobservable (Wooldridge 2010). This study reduced the selection on unobservable by conditioning on a set of covariates based on Equation (5). The second assumption opined that by conditioning on a set of covariates, there is a positive likelihood of getting treatment by each individual which is called the “overlap assumption.” Consequently, we only satisfied the assumption that for each participating farmer in the sample we observe some non-participating farmers with common covariates. However, if the choice of the specification is sensitive to the estimators, then the overlap assumption is violated giving rise to vague estimates (Crump et al., 2009). The normalized differences for each covariate were used to address the overlap assumption (Imbens and Wooldridge, 2009) specified as:

$${\text{normdiff}}_{j}=\frac{\left({\bar{X}}_{1j}-{\bar{X}}_{0j}\right)}{\sqrt{{\hat{\sigma }}_{ij}^{2}+{\hat{\sigma }}_{0j}^{2}}}$$

Where; ${\bar{X}}_{1j}$ and ${\bar{X}}_{0j}$ represent the means for the covariate j for the participants and non-participants; ${\hat{\sigma }}_{ij}^{2}$ and ${\hat{\sigma }}_{0j}^{2}$ represent the estimated standard deviations.

We further employed propensity score matching as a robustness check based on Imbens and Wooldridge (2009) to estimate the treatment effects. The propensity scores matching focus on matching the propensity scores of missing data. We employed the nearest neighbor, kernel, and caliper matching algorithms of PSM.

Moreover, we delved into the impact of specific SIPMA interventions on the outcomes (yield, household farm income, food expenditure and non-food expenditure) using the multivalued inverse probability weighted regression adjustment (MIPWRA). The most participated SIPMA interventions are input credit provision, structured market and entrepreneurial training. Therefore, a farmer has eight mutually exclusive participation choices of SIPMA interventions such as *non-participation* (I_0_S_0_E_0_), *input credit provision* (I_1_S_0_E_0_), *structured market* (I_0_S_1_E_0_), *entrepreneurial training* (I_0_S_0_E_1_), *input credit provision and structured market* (I_1_S_1_E_0_), *input credit provision and entrepreneurial training* (I_1_S_0_E_1_), *structured market and entrepreneurial training* (I_0_S_1_E_1_), and *input credit provision, structured market, and entrepreneurial training* (I_1_S_1_E_1_). Each farmer chooses one of the eight possible combinations to achieve optimum benefit. We estimate the multinomial logit to obtain the propensity scores for participation in SIPMA interventions. Hence, we compute for each combination using the inverse probability of treatment weights. In the second stage, the outcome model is computed based on the weighted regression from estimated weights for each treatment. To correct for any potential error from the computed propensity scores, the generalized method of moments (GMM) is used. The ATT of MIPWRA was computed by calculating the inverse of the treatment probability weights for farmers who participated in any of combinations of SIPMA interventions and expressed as:

(9) $$ATT_{y\tilde{i},\tilde{y}}=E\left[\left(H_{y\tilde{i}}-H_{1i}\right)|y=\vec{y}\right]$$

where; $y\tilde{i}$ represent the *i*th select farmer’s possible outcome from the yth treatment combinations, $\tilde{y}$ represent the treatment status of the treated possible outcome, $y=\tilde{y}$ represent the restriction for farmers who received treatment only $\vec{y}$ and 0 is possible treatment status of each outcome for control group.

## 3 Results and discussion

### 3.1 Descriptive results

Table 2 presents the sociodemographic characteristics of research participants. The results show that overall maize production is dominated by men (87 %) with higher proportion of non-participant (88.3 %) than participants (85.3 %). Maize farmers are economically active; having an average age of 47.6 years and was insignificant among the two groups. More than half of the non-participant farmers (51.3 %) were indigenous. Overall, most of the farmers (67.5 %) engaged in off farm activity and varied significantly between the two groups. This result is similar with Babatunde and Qaim (2018) who posit that farmers with high-income could assemble productive resources and diversify more easily than low-income farmers. A typical farmer has completed 8 years of education which was significantly higher for participants (8.4 years) than non-participants (7.6 years). This implies that generally, most of the maize farmers in Ghana had completed basic education considerably. In all, participants had obtained significantly higher years of experience in maize production (23 years) compared to non-participants (20 years). This indicates that experienced farmers could easily adopt innovate ideas acquired through extension advice and Farmer Based Organization trainings to improve crop productivity.

**Table 2:** Socioeconomic characteristics of maize farmers.

Variable	Participants (N = 212)	Non- Participants (N = 265)	Overall (N = 477)	t-stat
Age (Years)	47.7 (11.74)	47.5 (9.8)	47.6 (10.7)	−0.37
Sex (Male)^a^	0.853 (0.35)	0.883 (0.32)	0.87 (0.33)	0.34
Years of schooling	8.4 (5.1)	7.6 (4.3)	8.0 (4.8)	1.63
Residence status (indigene)^a^	0.476 (0.49)	0.513 (0.50)	49.6 (5.0)	1.38
Off farm activity^a^	0.726 (0.44)	0.633 (0.48)	0.675 (0.46)	−3.72∗∗∗
Maize experience (Years)	22.60 (12.8)	20.20 (12.2)	21.30 (12.5)	−3.51∗∗∗
Farm size (acre)	5.4 (2.53)	4.35 (2.86)	4.85 (3.16)	−4.08∗∗∗
Number of plots (N)	1.71 (1.31)	1.45 (0.82)	1.56 (1.07)	−4.43∗∗∗
Land ownership^a^	0.81 (0.38)	0.524 (0.49)	0.654 (0.47)	−12.08∗∗∗
Extension contacts^a^	0.608 (0.48)	0.566 (0.49)	0.584 (0.49)	−1.61
Distance to extension agent (km)	3.94 (1.39)	5.93 (2.35)	5.11 (1.35)	−5.81∗∗∗
Credit access^a^	0.875 (0.33)	0.594 (0.49_	0.751 (0.43)	−6.43∗∗∗
Market information^a^	0.381 (0.41)	0.240 (0.44)	0.490 (0.42)	4.60∗∗∗
Market distance (km)	3.10 (1.09)	5.23 (2.07)	4.34 (1.14)	3.63∗∗∗
FBO membership^a^	0.623 (0.48)	0.491 (0.50)	0.549 (0.49)	−5.03∗∗∗

a Figures are in percentages. Figures in parenthesis (.) are standard deviation. The asterisks, ∗, ∗∗ and ∗∗∗ denote that the differences in means across the treatment groups are significant level at 1 %, 5 % and 10 %, respectively. † = binary variable.

Overall, farmers cultivated an average of 4.8 acres of maize employing a mean of 1.6 plots. This was significantly higher for participants (2 plots) than non-participants (1 plot). Furthermore, about 65.4 % of the farmers, are landowners and varied considerably between two groups. This is consistent with Aschalew (2020) who posited that farmer with higher land size can considerably to expand maize production. The majority of the participants (60.8 %) had contact with extension agents higher than non-participants (56.6 %) and this had significant difference among the two groups. The high extension contacts suggest that farmers can improve yield through extension advice and supports. On average, farmers covered about 5.11 km to extension office and had significant difference between the two groups, thus, 3.9 and 5.9 km for participants and non-participants, respectively.

About 62.3 % and 49.1 % of the participants and non-participants were members of FBOs, respectively. This implies that FBO membership support farmers with basic technical knowledge and farm inputs to enhance maize productivity. In general, only 38.1 % of participants and 24 % of non-participant farmers had access to market information. Ownership of communication equipment (e.g., radio, television, etc.) among farmers may facilitate access to information regarding policy interventions that target farmers and their operations (Azumah and Zakaria, 2019). Two thirds of the farmers had access to credit for maize production comprising 87.5 % and 59.4 % for participants and non-participants, respectively. Consistent with previous research, access to credit has been found to stimulate farmers to increase crop production through increased access to timely supply of production inputs (Houeninvo et al., 2020; [26]). Furthermore, the average distance a participant maize farmer travelled to the nearest market is significantly lower (about 3.1 km) compared with that of non-participants who travelled about 5.23 km.

### 3.2 Econometric results

#### 3.2.1 Determinants of impact of participation on yield and household welfare

We analyzed the determinants of impact of participation on yield and welfare using the inverse probability weighted regression adjustment (IPWRA). As discussed previously, the IPWRA involved two stages, thus IPW and RA. According to Takahashi and Barrett (2013), the only purpose of propensity score estimation is to find a way to balance the observed covariates between participants and non-participants. As no causal interpretation will be drawn, the findings in Table 3 present the probit estimates of determinants of SIPMA intervention participation. The Wald test results for testing the hypothesis that β = 0 was statistically significant at 1 %, indicating that the explanatory variables influenced the likelihood of participation in the SIPMA intervention jointly.

**Table 3:** Probit estimates for determinants of participation in SIPMA interventions.

Variables	δy/δx	Robust S.E
Age	−0.009^b^	0.004
Sex (male)	0.176^c^	0.090
Residential status	−0.061	0.068
Years of schooling	0.017^b^	0.008
Off-farm activity	−0.525^a^	0.132
Maize experience	0.529^a^	0.109
Household size	−0.115	0.125
Farm size	0.083	0.103
Number of Plots	0.137^a^	0.040
Land ownership	0.148^b^	0.068
Market information	0.412^a^	0.137
Credit access	−0.435^a^	0.144
Extension contacts	0.191^b^	0.073
Ln Distance to extension office	−0.494^a^	0.183
Ln Market distance	−0.192^a^	0.035
FBO membership	0.288^b^	0.119
Constant	0.293^b^	0.123
Number of Observations	477	
Pseudo r-squared	0.384	
Wald X^2^	308.26	
Prob > chi2	0.000	

SE is Standard errors.

a p < 0.01.

b p < 0.05.

c p < 0.1.

Younger farmers are more likely to engage in the SIPMA intervention. Younger farmers are generally more eager to adopt new technologies than their elder counterparts (Jaafar et al., 2015; [26]). However, Awotide et al. [37] and Oladejo et al. [38] argue that age and participation in agricultural projects have no significant relationship. Gender has a negative effect on participation, indicating that male rural farmers in Ghana are more likely to participate in the SIPMA intervention than their female counterparts due to greater access to productive resources. This finding supports Nxumalo and Oladele's [39] conclusion that more male farmers are more likely to participate in agricultural projects. At a 5 % level of significance, education was significant and positive. Farmers with a higher education level are more likely to increase participation because they can interpret the benefits of the SIPMA intervention and make informed decisions. Baffoe et al. [40] posits that education status had significant effect on agricultural projects. Maize farmers with greater experience are more likely to participate in SIPMA activities.

Long years of maize cultivation raise farmers' awareness and facilitate their adoption of productive activities. Furthermore, the positive influence of plot number indicates that the number of plots owned by farmers encourages participation in the SIPMA activity, indicating that farmers tend to use other available plots to increase maize production. The positive effect of maize-farming household members suggests that additional household members are more likely to participate in SIPMA activities during the production period because they are more likely to support maize production activities. Farmers who engage in off-farm activity are less likely to participate in SIPMA, according to the off-farm activity variable, which was negative and statistically significant at 1 %. Generally, farmers are burden with activities other than farming due to high income earned to support basic needs of the household. This result supports Rakotoarisoa and Kaitibie's (2019) conclusion that there is a significant positive relationship between off-farm activity and participation in agricultural activity. Additionally, land ownership influenced SIPMA intervention participation positively. Farmers who own land are more likely to participate in SIPMA, and their likelihood of participation is significant at 5 %, indicating that farmers could release additional land to engage in SIPMA activities and increase land productivity as a result.

Extension contacts influenced SIPMA intervention participation significantly and positively. Farmers are more likely to adopt appropriate and improved production and marketing techniques through frequent extension services. However, greater distances to extension tend to decrease participation. This supports Eneyew's [41] assertion that there is a significant negative relationship between distance and participation. The negative effect of credit access and its significant influence on participation in the SIPMA project indicate that farmers are less likely to participate in the SIPMA project when they have approximately four times less access to credit, which prevents them from acquiring productive resources. However, Oladejo et al. [38] found no correlation between credit access and agricultural project participation.

We discuss the second stage of the IPWRA model, i.e., the determinants of participation's impact on yield and household welfare. Imbens and Rubin (2010) suggest that normalized differences as presented in Table 4 indicates that an absolute value of 0.25 or above should raise red flags to evaluate the overlap assumption. Table 4 reveals that only three of the normalized differences are greater than the absolute value of 0.25, indicating that the impact equation in section 3 can be used to calculate the ATT results. In this paper, income, food expenditure, and non-food expenditure are used as indicators of welfare. Table 5 displays the IPWRA's estimates of the impact of participation on maize yield, income, food, and non-food expenditures for both participants and non-participants. The results indicate that the impact varies significantly between participants and nonparticipants.

**Table 4:** Normalized differences addressing overlap assumption.

Variables	Participants	Non-participants	Difference normalized
	Mean	Mean	
Age	47.8	47.5	0.04
Sex (male)	0.388	0.491	0.01
Residential status	0.476	0.513	0.03
Years of schooling	8.4	7.6	−0.01
Off-farm activity	0.726	0.633	0.22
Maize experience	22.6	20.2	**0.32**^a^
Household size	6.6	6.5	0.06
Farm size	5.5	4.3	0.16
Number of Plots	2	1.5	0.02
Land ownership	0.821	0.525	−0.21
Market information	0.821	0.562	**0.27**^a^
Credit	0.875	0.594	−0.09
Extension contacts	0.792	0.668	0.21
Ln Distance to extension office	4.4	7.2	0.02
Ln Market distance	5.6	8.0	0.09
FBO membership	0.623	0.491	**0.26**^a^

Note.

a indicate difference of more than 0.25.

**Table 5:** Inverse probability weighted regression adjustment (IPWRA) estimates for the determinants of farmers’ welfare.

Variables	Yield		Household Farm Income		Food Expenditure		Non-Food Expenditure	
	Participants	Non-Participants	Participants	Non-Participants	Participants	Non-Participants	Participants	Non-Participants
Age	0.033	0.519	0.507∗∗	0.143∗∗∗	−0.026	0.320	−0.168∗∗∗	0.473∗
	(0.148)	(0.365)	(0.228)	(0.045)	(0.223)	(0.371)	(0.061)	(0.251)
Sex (male)	0.240∗∗∗	0.540∗∗∗	0.139	0.560∗∗∗	−0.069	−0.036	0.278	−0.102
	(0.078)	(0.169)	(0.120)	(0.175)	(0.103)	(0.202)	(0.237)	(0.189)
Education	−0.030	−0.228∗∗	0.304∗∗∗	0.023	0.080	0.146∗	−0.104	−0.183∗
	(0.037)	(0.099)	(0.063)	(0.095)	(0.068)	(0.075)	(0.124)	(0.094)
Residence status (indigene)	0.088	−0.158	−0.087	−0.390∗∗∗	0.323∗∗∗	−0.046	0.001	−0.159
	(0.054)	(0.112)	(0.083)	(0.131)	(0.078)	(0.091)	(0.144)	(0.142)
Off farm activity	0.134∗∗	−0.200∗	0.395∗∗∗	0.412∗∗∗	−0.075	0.338∗∗∗	−0.677∗∗∗	0.250∗
	(0.059)	(0.102)	(0.088)	(0.143)	(0.082)	(0.111)	(0.153)	(0.142)
Experience	0.030	0.287∗∗	0.296∗∗∗	0.167	0.385∗∗∗	0.346∗∗∗	0.055	0.210∗∗
	(0.058)	(0.117)	(0.084)	(0.137)	(0.079)	(0.107)	(0.162)	(0.084)
Household size	0.129∗∗∗	0.116	0.008	−0.454∗∗∗	0.090	−0.069	0.656∗∗∗	−0.221∗∗
	(0.041)	(0.112)	(0.104)	(0.115)	(0.058)	(0.101)	(0.176)	(0.097)
Farm size	0.129∗∗∗	−0.238∗∗	0.058	−0.022	0.124∗	0.309∗∗∗	−0.316∗∗	0.359∗∗∗
	(0.042)	(0.103)	(0.061)	(0.105)	(0.069)	(0.101)	(0.139)	(0.102)
Number of plots	0.087∗	−0.057	−0.103	0.883∗∗∗	0.122	−0.503∗∗∗	−0.787∗∗∗	−0.070
	(0.046)	(0.202)	(0.072)	(0.190)	(0.081)	(0.169)	(0.237)	(0.114)
Market information	0.069∗∗∗	0.030	−0.080	−0.654∗∗∗	0.255∗∗	−0.141	0.582∗∗∗	−0.118
	(0.016)	(0.178)	(0.085)	(0.188)	(0.105)	(0.107)	(0.219)	(0.155)
FBO participants	0.175∗∗∗	0.095	0.262∗∗∗	−0.203∗	0.140∗	−0.125	−0.156	0.066
	(0.041)	(0.101)	(0.073)	(0.120)	(0.075)	(0.092)	(0.165)	(0.131)
Credit	0.109∗∗	−0.429∗∗∗	0.371∗∗∗	−0.321∗	0.355∗∗∗	−0.241	−0.838∗∗∗	−0.043
	(0.050)	(0.141)	(0.087)	(0.166)	(0.089)	(0.164)	(0.190)	(0.114)
Ln Distance to extension office	−0.151∗∗	−0.989∗∗∗	−0.995∗∗∗	0.291	−0.104	−0.703∗∗∗	−0.078	−0.125
	(0.062)	(0.238)	(0.093)	(0.224)	(0.091)	(0.208)	(0.286)	(0.145)
Ln Market distance	0.014	−0.173∗∗∗	0.150∗∗∗	−0.102∗∗	0.028∗	−0.073∗	−0.083	−0.002
	(0.010)	(0.054)	(0.015)	(0.049)	(0.015)	(0.043)	(0.071)	(0.024)
Constant	0.713∗∗∗	0.482∗∗∗	0.308∗∗∗	0.162	0.706∗∗∗	0.449∗∗∗	0.147∗∗∗	0.737∗∗∗
	(0.049)	(0.127)	(0.078)	(0.147)	(0.075)	(0.118)	(0.019)	(0.098)
Balancing test after propensity score reweighting:								
Over identification test for covariate balance			χ^2^ = 21.8; P> χ^2^ = 0.335					

Age has a significant positive effect on maize yield and non-food expenditure for both participants and non-participants. This is because, as farmers age, they gain experience and put their acquired knowledge into practice, thereby increasing their maize yields. Older farmers increase non-food expenditure to build household wealth because they require less food expenditure. The findings support Bellemare's (2012) claim that older farmers are more likely to participate in the market. However, age had a negative effect on farm income, indicating that older farmers are more prone to illness and stress, and thus end up spending farm income on drugs. Furthermore, older farmers were found to spend less money on food than younger farmers. In general, older farmers in rural areas spend less money on food because they rely on their farm products.

Sex, especially male, variable shows positive impact on both food and non-food consumption expenditures for participants. This means expenditures, generally increases among male-headed household than female-headed counterparts. Similar to Dzanku [42], however contradicts with Kpoor [43] reported that consumption expenditure for female-headed farmers well-heeled than male-headed counterparts in Ghana. In addition, male farmer shows positive impact on maize yield for non-participants indicating that male farmers are usually decisions makers in terms of access and control of productive resources, hence, enhance maize yield.

Education plays a substantial role in determining maize yield and household food expenditures. The positive coefficient of education implies that an additional year of schooling increase maize yields for participants. Educated farmers accumulate good knowledge of agricultural practices and may tend to adopt to double yields [8,26]. However, educated farmers show negative significant impact on food expenditure among participation. Education is not entirely a determining variable in food consumption expenditure rather the available income determines food consumption expenditure [37]. Educated households tend to reduce food consumption expenditure compared to uneducated households. The idea that education is important in helping farmers to reduce food consumption expenditures rely mostly on their farm produces. Educated farmers are expected to have higher incomes but turn up to limit household food expenditure. Similar findings with Gebrehiwot et al. [44] and Tigga [45] in Ethiopia.

Household size had negative significant impact on food expenditure for participants, suggesting that small household size tend to spend less on food consumption. Additional household member in maize activity impact food expenditure positively and significantly. Consequently, household member in maize activity had a positive significant impact both on maize yield and income among participants. For instance, an additional household member in maize activity tend to support production activities on time and could join the SIPMA intervention to access farm inputs to support maize production. A member may assist in farming operations such as fertilizer application, planting, agrochemical application etc., hence, add up to farm household expenditure. This agrees with Abdissa et al. [46] and Tigga [45] in Ethiopia. However, there is a positive and significant impact of household size on both food and non-food consumption expenditures among non-participants. For instance, larger households are more endowed with labor supports in farming activities [8] which tend to increase food and non-food items.

Experience in maize production had a negative impact on household non-food expenditure among participants, most likely because farmers with extensive knowledge may prefer to achieve high maize yield by reinvesting farm income in production activities rather than spending it on other items. The land ownership variable had a significant negative impact on maize yield and income. Farmers who operated under various forms of land ownership (e.g., sharecropping, rented, etc.) could reduce maize yield and income due to the division of maize outputs, thereby affecting their incomes. Furthermore, land ownership has a negative impact on both groups' food expenditure. This is because farmers who sharecrop prefer to spend less on food to save for future land purchases. Farmers who engage in off-farm activity generally increase food expenditure for participants; thus, off-farm activity significantly increases household food expenditure by 40 %. Farmers engage in off-farm work to meet household basic needs. Nonetheless, for non-participants, off-farm activity had a negative and significant impact on maize yield, indicating that high-income farmers may tend to diversify their income sources and venture into off-farm activities [47,48].

Large farms and a greater number of plots increase maize yield for participants because farmers have greater access to farm inputs from the SIPMA intervention to expand production, thereby increasing maize yield. This is consistent with Chen et al. [49], who discovered that farm size has a positive effect on yield in China. The positive relationship between the size of maize farms and non-food expenditures suggests that farm size as a non-food item may serve as a proxy for diversification to expand maize production activities. However, the number of plots and the size of the farm have a negative and significant impact on both groups' income and non-food expenditures. This is because farmers who invest in an additional plot or land for maize production decrease their income and non-food expenditures.

The balancing test following reweighting of propensity scores is also depicted in Table 5. The results indicate that the null hypothesis of covariate balance cannot be rejected, as there is no evidence that the covariates remained unbalanced after propensity score reweighting. Consequently, we can proceed with estimating the ATTs for our outcome variables.

#### 3.2.2 Impact of participation in agricultural intervention on yield and household welfare

The estimates for the average treatments effect on treated (ATET), which show the impact of SIPMA participation on maize yield, farm income, food, and non-food expenditures, are presented in Table 6. The ATET estimates account for selection bias arising from the fact that participants and non-participants may be steadily different. Results show that participation signiﬁcantly increases yields and incomes. Consequently, the causal effect of participation in SIPMA intervention is 317.44 kg per acre, representing a 36.5 % increase in yields. Also, the participation in SIPMA increased income by 39.1 %. Household food expenditure reveal that the casual effect of participation in the SIPMA is GHS624.44, indicating 13.1 % decrease in food expenditures. Lastly, about 5.7 % increase in non-food expenditure because of participation. These ﬁndings are consistent with the view that participation in agricultural projects can improve farm yield and household farm incomes as well as food and non-consumption patterns [7,48,50].

**Table 6:** Inverse probability weighted regression adjustment (IPWRA) estimates of Average treatment effects.

Outcome variables	Participation		Average treatment effects (ATT)
	Participants	Non-Participants	
Yield (kilogram/acre)	1185.32	868.08	317.24∗∗∗ (113.44)
Household farm income (in GHS)	2797.19	2011.35	785.84∗∗∗ (259.09)
Food expenditure (in GHS)	4158.88	4783.32	−624.44∗∗∗ (198.82)
Non-food expenditure (in GHS)	7436.02	7037.99	398.03∗∗∗ (118.88)

Note: Robust standard errors in parentheses. ∗∗∗, ∗∗, and ∗ indicates significance at 1 %, 5 %, and 10 % levels, respectively. 120 kg = 1 bag of maize.

#### 3.2.3 Factors influencing the choice of specific SIPMA interventions

Table 7 shows the multinomial logit estimates of factors influencing farmers’ decision to choose different SIPMA interventions. We used farmers who did not participate in any of the specific SIPMA interventions as the reference group (I_0_S_0_E_0_). The Wald test result [X^2^ = 339.22; p = 0.000] rejects the hypothesis that all regression coefficients are jointly equal to zero. The findings demonstrate that the estimated coefficients across the various SIPMA interventions vary significantly. The key variables influencing the choice of specific SIPMA interventions are education, extension contact, distance to market and interest free credit. Our results indicate that educated farmers are more likely to combine input credit provision, structured market and entrepreneurial training (I_1_S_1_E_1_) to improve crop yield. Improved education enables farmers to understand the advantages of SIPMA interventions and encourages them to participate, particularly in productivity-management-improving interventions like input credit provision and entrepreneurial training. This corroborates with previous findings [[51], [52], [53]]. For instance, Orinda [52] asserted that education may enable farmers to make effective decisions and act as early participants to benefit from new agricultural interventions in Kenya.

**Table 7:** Multinomial logit estimates of the factors influencing the choice of specific SIPMA interventions.

Variables	I_1_S_0_E_0_	I_0_S_1_E_0_	I_0_S_0_E_1_	I_1_S_1_E_0_	I_1_S_0_E_1_	I_0_S_1_E_1_	I_1_S_1_E_1_
	δy/δx	δy/δx	δy/δx	δy/δx	δy/δx	δy/δx	δy/δx
Age (years)	−0.043	−0.109	−0.162∗∗∗	0.127	0.173	0.142	0.262
	(0.181)	(1.712)	(0.057)	(0.156)	(0.164)	(0.167)	(0.169)
Sex (married)	0.147∗	0.571	0.113	0.860	0.647	0.623	0.771
	(0.087)	(0.703)	(0.837)	(0.667)	(0.708)	(0.727)	(0.718)
Marital status (married)	−0.140	−0.653	−0.908	−0.374	−0.661	0.456	−0.721
	(0.986)	(0.941)	(0.964)	(0.894)	(0.921)	(1.027)	(0.961)
Education (years)	−0.128	0.474∗∗∗	0.498	0.515	0.354	0.367	0.664∗∗∗
	(0.403)	(0.038)	(0.448)	(0.361)	(0.394)	(0.390)	(0.289)
Experience (year)	0.641	0.637	0.109∗	−0.567	0.108∗	−0.582	−0.123∗∗
	(0.636)	(0.654)	(0.062)	(0.575)	(0.059)	(0.614)	(0.060)
Household size	0.105	−0.135	−0.114	0.030	0.101	0.123	0.617
	(0.065)	(0.562)	(0.589)	(0.534)	(0.563)	(0.583)	(0.602)
Farm size (acre)	0.177∗∗∗	0.141	0.226	0.237	−0.165	0.140	0.180∗∗∗
	(0.047)	(0.417)	(0.443)	(0.397)	(0.432)	(0.428)	(0.045)
Land ownership	−0.847	0.486	0.961	−0.177	0.075	0.507	0.394
	(0.558)	(0.585)	(0.677)	(0.522)	(0.560)	(0.596)	(0.598)
Off farm activity	−0.185	0.244	−0.122∗∗	0.203	−0.458	0.014	−0.604
	(0.584)	(0.585)	(0.061)	(0.530)	(0.552)	(0.584)	(0.573)
Distance to farm (km)	−0.106∗∗	−0.581	0.858	−0.509	0.706	0.132∗	−0.706
	(0.047)	(0.496)	(0.560)	(0.399)	(0.436)	(0.068)	(0.472)
FBO membership	0.340	0.154∗∗∗	0.586	0.110∗∗	0.570	0.110∗∗	0.838
	(0.517)	(0.049)	(0.544)	(0.046)	(0.496)	(0.050)	(0.514)
Extension contact	−0.334	0.114∗∗	0.161∗∗∗	0.619	0.870∗	0.147∗∗∗	0.1536∗∗∗
	(0.537)	(0.051)	(0.060)	(0.475)	(0.526)	(0.053)	(0.057)
Market information	0.364∗∗∗	−0.111	−0.274	0.804	0.136∗	−0.181∗∗	0.701
	(0.094)	(0.077)	(0.836)	(0.715)	(0.075)	(0.082)	(0.762)
Distance to market (km)	−0.839∗	−0.375	−0.476	−0.154	−0.516	−0.138∗∗	−0.633
	(0.460)	(0.460)	(0.518)	(0.383)	(0.422)	(0.063)	(0.449)
Distance to the nearest training center (km)	−0.132∗	−0.532	−0.125	−0.591	−0.819	−0.142∗	−0.506
	(0.075)	(0.767)	(0.081)	(0.720)	(0.764)	(0.077)	(0.835)
Access to hybrid seed	0.141∗∗	0.083	0.850	0.181	0.362	−0.183	−0.119
	(0.061)	(0.576)	(0.630)	(0.545)	(0.578)	(0.585)	(0.599)
Pest disease stress	0.134	−0.233	−0.530	−0.037	−0.113∗	−0.155∗	−0.550
	(0.622)	(0.596)	(0.640)	(0.556)	(0.058)	(0.059)	(0.601)
Poor soil and water conservation	0.696	0.139	−0.396	0.242	−0.065	0.025	0.057
	(0.743)	(0.719)	(0.836)	(0.681)	(0.719)	(0.731)	(0.754)
Interest free credit (yes)	0.1187∗	0.176∗∗	0.146∗	0.226∗∗∗	0.157∗∗	0.847	0.267∗∗∗
	(0.070)	(0.077)	(0.083)	(0.067)	(0.070)	(0.805)	(0.074)
Constant	0.170	−0.265	−0.402	−0.518	−0.294	−0.221	−0.834
	(0.602)	(0.576)	(0.596)	(0.528)	(0.551)	(0.572)	(0.576)
Observations	477						
Wald chi-square	339.22∗∗∗						
LR (140)	516.70∗∗∗						
Pseudo R^2^	0.1876						
Log likelihood	111.903						

Note: Robust Standard errors are in parenthesis. I_0_S_0_E_0_ used as based category. ∗∗∗, ∗∗ and ∗ are 1 %, 5 % and 10 %, respectively.

Farmers with access to extension contact are more likely to participate in specific SIPMA interventions, such as structured market alone (I_0_S_1_E_0_), entrepreneurial training alone (I_0_S_0_E_1_) and all three SIPMA interventions (I_1_S_1_E_1_). Participation in agricultural programs mostly by extension contact via extension agents advocates the essence of participation modern agricultural programs to improve crop production. For example, Suvedi et al. [54] opined that farmers' contact with extension agents increase participation in farm related inputs and training on good farm practices in Nepal. Also, Nakano et al. [55] found that farmer-to-farmer training through extension programs improves farmers' participation in farm-productive resources such as fertilizer, and improved seeds in Tanzania.

The positive significant coefficients of interest free credit suggest that farmers are more likely to participate in at least one of the combinations except structured market and entrepreneurial training (I_0_S_1_E_1_). Fenger et al. [56] indicated that an increase in yield and quality of agricultural products may result from agricultural programs which may also give farmers better access to resources like inputs, education, training and credit. The negative coefficient of distance from homestead to the nearest market indicates that farmers are less likely to combine structured market and entrepreneurial training (I_0_S_1_E_1_). This implies that additional kilometer, discourages participation due to higher transaction costs. Our findings corroborate with Anang and Amikuzuno [57] found that extra market distance is predicted to increase transaction costs associated which is likely to reduce the participation in agricultural programs.

#### 3.2.4 Impact of specific SIPMA interventions on yield and welfare

We further estimated the impact of key SIPMA interventions on smallholder farmers’ welfare (Table 8). The key specific interventions are input credit provision, structured market, and entrepreneurial training. Using the MIPRA model, the results reveal that participation in specific SIPMA interventions improve yield, farm income, food expenditure and non-food expenditure in Ghana. A combination I_1_S_1_E_1_ indicates that smallholder farmers obtain the highest yield, farm income and non-food expenditure. Adoption of entrepreneurial training alone gives lower yield and income compared to other combinations. The adoption of entrepreneurial training alone may not effectively address the resource constraints faced by farmers. Limited availability of quality inputs, machinery, and credit can hinder farmers from fully maximizing their yield potential. Furthermore, while entrepreneurial training equips farmers with valuable business skills, the absence of adequate market linkages can pose challenges in finding profitable outlets for their produce. Moreover, difficulties in accessing markets, negotiating fair prices, and establishing reliable supply chains can significantly impact farmers' income levels. On average, participation in SIPMA interventions lead to better improvement in yield, income, food expenditure and non-food expenditure. These findings are consistent previous studies ([13]; Biru et al., 2020; Prah et al., 2023). Prah et al. (2023) found that participation in agricultural interventions improves maize productivity and productivity in Ghana. Iddrisu et al. [30] found also that participation in UTZ-RA voluntary cocoa certification scheme enhances smallholder farmers welfare in Ghana.

**Table 8:** Multivalued results of average treatment effect for specific SIPMA interventions.

Combinations		Yield (kilogram/acre)	Farm income (GHS)	Food expenditure (GHS)	Non-Food expenditure (GHS)
I_1_S_0_E_0_	ATT	1999.5^a^ (169.29)	2170.85^a^ (176.66)	157.01^a^ (9.51)	1948.80^a^ (121.38)
I_0_S_1_E_0_	ATT	1925.77^b^ (748.45)	4224.49^a^ (634.92)	66.32^a^ (7.648)	1868.31^a^ (212.69)
I_0_S_0_E_1_	ATT	2119.06^a^(130.38)	1749.31^b^ (683.55)	190.42^a^ (18.93)	2365.94^a^ (186.74)
I_1_S_1_E_0_	ATT	2530.19^a^ (90.15)	3592.76^a^ (175.97)	132.92^a^ (6.86)	1659.28^a^ (85.99)
I_1_S_0_E_1_	ATT	2235.93^a^ (106.10)	3493.36^a^ (209.09)	144.39^a^ (7.83)	2102.32^a^ (98.30)
I_0_S_1_E_1_	ATT	2683.80^a^ (415.97)	2987.94^a^ (193.22)	57.70^a^ (6.37)	3005.19^a^ (654.32)
I_1_S_1_E_1_	ATT	2776.90^a^ (149.78)	4843.29^a^ (411.67)	145.95^a^ (15.12)	3190.88^a^ (589.45)

Note: Robust standard errors in parenthesis.

a p < 0.01.

b p < 0.05, ∗p < 0.1. 120 kg = 1 bag of maize.

### 3.3 Robustness check

The propensity score matching (PSM) and Imben’s exogeneity analysis were used as robustness check of the IPWRA model. The results of the PSM and Imben’s exogeneity are presented in Table 9, Table 10, respectively. After controlling for the endogeneity, the results confirm that SIMPA participation enhanced smallholder maize farmers welfare in Ghana (Table 10). The impact of participation in SIPMA interventions using the various algorithm of PSM to check the robustness. The results show that participation led to 9.38–14.6 % increase in yield, GHS3071.87 to GHS4349.46 increase in household farm income, 15.38%–19.9 % reduction in food expenditure, and 11.1–16.9 % increase in non-food expenditure (Table 9). This implies that participation in SIPMA interventions is important to increasing smallholder maize farmers’ welfare. The findings support the findings of Iddrisu et al. [30] who found the participation in agricultural intervention such as UTZ-RA voluntary certification program has a positive significant impact on yield and income. Similarly with findings of Awotide et al. [37] found agricultural technology has positive impact on asset ownership in Nigeria.

**Table 9:** Average treatment effect from the propensity score matching.

Outcome	ATT
Yield:	
Nearest neighbor matching	938.78∗∗∗ (347.53)
Radius matching	1059.86∗∗∗ (106.14)
Kernel based matching	1460.95∗∗∗ (190.11)
Household Farm Income:	
Nearest neighbor matching	3066.02∗∗∗ (1014.44)
Radius matching	4349.46∗∗∗ (390.39)
Kernel based matching	3071.87∗∗∗ (512.70)
Household Expenditure:	
Nearest neighbor matching	−1811.14∗∗∗ (107.88)
Radius matching	−1538.13∗∗∗ (117.39)
Kernel based matching	−1994.05∗∗∗ (308.50)
Household Non-Food Expenditure:	
Nearest neighbor matching	1433.55∗∗∗ (109.97)
Radius matching	1118.47∗∗∗ (108.78)
Kernel based matching	1691.44∗∗∗ (296.96)

Note: ATT – Average treatment effect; GHS, Ghana cedis. One bag is equivalent to 120 kg ∗∗∗significant at 1 %, ∗∗significant at 5 %, ∗significant at 10 %.

**Table 10:** Imben’s robustness check.

Outcome	ATT	*Test of endogeneity*
		H_0_ = treatment and outcome unobservables are uncorrelated
Yield	969.53∗∗∗ (69.73)	Chi2 = 16.91 (p = 0.000)
Household Farm Income	3172.96∗∗∗ (187.83)	Chi2 = 25.03 (p = 0.000)
Household Expenditure	−1764.06∗∗∗ (107.88)	Chi2 = 30.32 (p = 0.000)
Household Non-Food Expenditure	1094.53∗∗∗ (65.10)	Chi2 = 8.09 (p = 0.0175)

Note: ATT – Average treatment effect; GHS, Ghana cedis. One bag is equivalent to 120 kg ∗∗∗significant at 1 %, ∗∗significant at 5 %, ∗significant at 10 %.

**Fig. 2:**
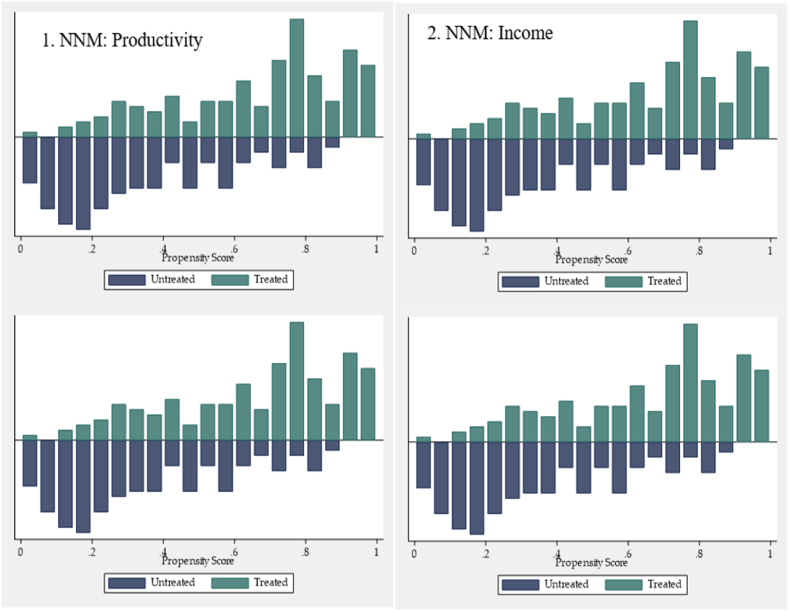
Histogram of propensity scores among participants and non-participants.

Source: Authors’ design, 2021.

Fig. 2 illustrated the visual presentation the distribution of propensity density scores for the participants and non-participants of the SIPMA intervention. The figure clearly indicates a difference between maize farmers who received treatment and those who did not, thus, those of SIPMA support and those not. Evidently, the figure shows relatively overlaps in the propensity scores distribution.

## 4 Conclusion and policy recommendations

This study examined the effect of participation in agricultural development interventions on maize yield and household welfare among smallholder maize farmers in Ghana. Using multivariate endogenous switching regression, inverse probability weighted adjustment, and propensity score matching, we estimated the factors influencing participation and impact on yield and household welfare. Farmers' participation in any of the SIPMA interventions –input credit, structured markets and enterprise development – is influenced by age, sex, education, off-farm activity, experience, household size, access to credit and land ownership, and innovative membership, according to our findings. Extension services and FBO membership play a significant role because both farmers and officers anticipate sharing and addressing participation challenges.

Our results accounted for sample selection bias because participation decisions of the outcome variables were estimated; consequently, participation in agricultural intervention in the case of the SIPMA might not have the same effect on non-participating smallholder farmers. The results of the average treatment effects model indicate that participation in the SIPMA intervention has a positive and significant impact on maize yield and household welfare indicators, including household income, household food expenditure, and non-food expenditure. For instance, participants in the SIPMA intervention have an advantage over non-participants in terms of increasing yield and gaining access to structured and ready markets. Due to access to mechanization services and input credits, the results suggest that participant farmers are more motivated to increase production. Farmers who have greater access to productive resources because of agricultural interventions are more likely to participate; therefore, access to productive resources plays a crucial role in determining participation.

On the effect of participation on maize yield, the results revealed a casual effect of approximately 36.5 %, indicating that SIPMA interventions in regions with low maize yield contribute significantly to productivity growth. In addition, income and non-food expenditure showed a casual increase effect of 39.1 % and 5.7 %, respectively, for participants. Nevertheless, food expenditures decreased by 13.1 %. Moreover, participation in certain SIPMA interventions suggests that participation improves the welfare of smallholder farmers in Ghana. Furthermore, the robustness check estimators produced similar impact results for smallholder farmers in terms of yield, income, food, and non-food expenditures. Given that our findings are related to other studies of agricultural interventions, it has the potential to significantly impact maize yield and household welfare among smallholder farmers (Hassin et al., 2019; [13,20]).

Our findings suggest that effective policy measures are needed to promote and scale up SIPMA intervention participation in other communities, as well as improve farmer information channels to learn more about the various interventions. Thorough research and investment to improve participation in all SIPMA activities would be necessary to improve production efficiency programmes that increase marketing, input credit, and participation in enterprise development. Given the positive significant influence of extension contact and farmer based organization, we recommend an investment in strengthening agricultural extension services and supporting farmer-based organizations to improve information-sharing and customize programs to meet the unique needs of diverse farmer profiles, accounting for factors such as age, gender, education, and resource access. Also, scaling up SIPMA interventions in low-yield regions can drive substantial productivity gains, leading to increased incomes and spending for participating farmers. Furthermore, the design, implementation, and evaluation of such interventions - SIPMA - should consider increased coordination among multisectoral actors such as MoFA, to build capacities of smallholder farmers, thereby increasing yields and farm income.

## Funding

This work was funded by the Aliance for. Green Revolution in Africa, Ghana by providing the support for the data collection. Grant Number :17994.

## CRediT authorship contribution statement

**Bright O. Asante:** Investigation, Formal analysis, Data curation, Conceptualization. **Stephen Prah:** Writing – review & editing, Writing – original draft, Investigation, Data curation, Conceptualization. **Omphile Temoso:** Methodology, Investigation, Funding acquisition, Data curation, Conceptualization. **Forster Boateng:** Writing – review & editing, Resources, Investigation, Funding acquisition. **Abubakar Gyinadu:** Writing – review & editing, Conceptualization.

## Data availability

Data will be made available upon request.

## Notes

Yield was measured as the quantity of maize harvested in kilogram per acre.

Farm income was measured as the amount of output harvested sold minus the total cost of production in Ghanaian cedis.

Food expenditure is the amount of food acquired during the year in Ghanaian cedis.

Non-food expenditure is the amount of non-food items during the year in Ghanaian cedis.

Input credit provision was captured as farmer’s access to fertilizers and improved seeds during maize production season.

Structured market was captured as farmer’s ability to access structured market for maize after harvesting.

Entrepreneurial training was measured as the access to entrepreneurial training during maize production season.

## Declaration of competing interest

The authors declare the following financial interests/personal relationships which may be considered as potential competing interests:Bright Owusu Asante reports administrative support and statistical analysis were provided by Kwame Nkrumah University of Science and Technology Department of Agricultural Economics Agribusiness and Extension. If there are other authors, they declare that they have no known competing financial interests or personal relationships that could have appeared to influence the work reported in this paper.

## References

[1] UNCTAD (2022). Food export restrictions hurt millions in least developed countries. https://unctad.org/topic/least-developed-countries/chart-march-to-june-2022.

[2] Pandey V.L., Dev M.S., Jayachandran U. (2016). Impact of agricultural interventions on the nutritional status in South Asia: a review. Food Pol..

[3] Asante I.K., Inkoom E.W., Ocran J.K., Akaba S. (2022). Sustainability analysis of programme interventions: the case of root and tuber improvement and marketing programme in the central region of Ghana. ADRRI Journal of Agriculture and Food Sciences.

[4] Atindana B., Amfo B. (2023). Maize farmers' access to input credit and training, technology adoption and productivity in Sissala West District a case study of Guohaballe Agriculture and Business Ventures. https://www.researchgate.net/publication/368645554_Maize_farmers'_access_to_input_credit_and_training_technology_adoption_and_productivity_in_Sissala_West_District_a_case_study_of_Guohaballe_Agriculture_and_Business_Ventures.

[5] Asante-Addo C., Mockshell J., Zeller M., Siddig K., Egyir I.S. (2017). Agricultural credit provision: what really determines farmers' participation and credit rationing?. Agric. Finance Rev..

[6] Adams A., Jumpah T.E., Shafiullah M. (2021). Agriculture technologies adoption and smallholder farmers welfare: evidence from Northern Ghana. Cogent Economic & Finance.

[7] Anang B.T., Bäckman S., Sipiläinen T. (2020). Adoption and income effects of agricultural extension in northern Ghana. Scientific African.

[8] Abdulai A., Huffman W. (2014). The adoption and impact of soil and water conservation technology: an endogenous switching regression application. Land Econ..

[9] Ocansey R.T.A., Nyawornota V.K., Adamba C., Tay D.A., Musah K., Nyanyofio O.C.N., Malete L., McCole D. (2023). Promoting development of entrepreneurial skills of youth in Ghana through a structured sport intervention program. Frontiers in Education.

[10] Osei C.D., Zhuang J. (2024). The effects of institutional supports on farm entrepreneurial performance: exploring the mediating role of entrepreneurial orientation. Sage Open.

[11] Kæmsgaard L., Schmidt P. (2017).

[12] Muluken G.W., Sassi M. (2020). Impact of agricultural interventions on food and nutrition security in Ethiopia: uncovering pathways linking agriculture to improved nutrition. Cogent Food Agric..

[13] Olagunju K.O., Ogunniyi A.I., Awotide B.A., Adenuga A.H., Ashagidigbi W.M. (2020). Evaluating the distributional impacts of drought-tolerant maize varieties on productivity and welfare outcomes: an instrumental variable quantile treatment effects approach. Clim. Dev..

[14] Ton G., de Grip K., Klerkx L., Rau M., Douma M. (2013).

[15] Michler J.D., Baylis K., Arends-Kuenning M., Mazvimavi K. (2019). Conservation agriculture and climate resilience. J. Environ. Econ. Manag..

[16] Scheiterle L., Birner R. (2018). Assessment of Ghana's comparative advantage in maize production and the role of fertilizers. Sustainability.

[17] Lajqi S., Thaqi M., Kaçiu K., Bytyqi H., Krasniqi B.A. (2017). Impact of agricultural intervention programs on income and employment: evidence from vegetable sector in kosovo. Ekon. Misao I Praksa Dbk..

[18] Nakano Y., Magezi E.F. (2020). The impact of microcredit on agricultural technology adoption and productivity: evidence from randomized control trial in Tanzania. World Dev..

[19] Karlberg L., Garge K.K., Barron J., Wani S.P. (2015). Impacts of agricultural water interventions on farm income: an example from the Kothapally watershed, India. Agric. Syst..

[20] Darko F.A., Palacios-Lopez A., Kilic T., Ricker-Gilbert J. (2018). Micro-level welfare impacts of agricultural productivity: evidence from rural Malawi. J. Dev. Stud..

[21] Roodman D., Morduch J. (2014). The impact of microcredit on the poor in Bangladesh: revising the evidence. J. Dev. Stud..

[22] Asfaw S., Kassie M., Simtowe F., Lipper L. (2012). Poverty reduction eﬀects of agricultural technology adoption: a micro-evidence from rural Tanzania. J. Dev. Stud..

[23] Bezu S., Kassie G.T., Shiferaw B., Ricker-Gilbert J. (2014). Impact of improved maize adoption on welfare of farm households in Malawi: a panel data analysis. World Dev..

[24] Kassie M., Marenya P., Tessema Y., Jaleta M., Zeng D., Erenstein O. (2018). Measuring farm and market level economic impacts of improved maize production technologies in Ethiopia: evidence from panel data. J. Agric. Econ..

[25] Manda J., Alene A.D., Gardebroek C., Kassie M., Tembo G. (2016). Adoption and impacts of sustainable agricultural practices on maize yields and incomes: evidence from rural Zambia. J. Agric. Econ..

[26] Etwire M.P., Dogbe W., Wiredu A.N., Martey E., Etwire E., Owusu R.K., Wahaga E. (2013). Factors influencing farmer's participation in agricultural projects: the case of the agricultural value chain mentorship project in the northern region of Ghana. J. Econ. Sustain. Dev..

[27] Laborde D., Bizikova L., Lallemant T., Smaller C. (2016). Ending hunger: what would it cost? IISD and IFPRI. https://www.iisd.org/sites/default/files/publications/ending-hunger-what-would-it-cost.pdf.

[28] Donkoh S.A., Eliasu A., Setsoafia E.D., Ansah I.G.K. (2016). Participation and output effect of a Block farm credit programme in selected districts of Northern Ghana. Agric. Finance Rev..

[29] Satapathy J., Nayak N.C., Mahakud J. (2020). Multidimensional impact of food security on household welfare: evidences from a household survey in three Indian states. Int. J. Soc. Econ..

[30] Iddrisu M., Aidoo R., Wongnaa C.A. (2020). Participation in UTZ-RA voluntary cocoa certification scheme and its impact on smallholder welfare: evidence from Ghana. World Dev.Perspect..

[31] Nechifor V., Ramos M.P., Ferrari E., Laichena J., Kihiu E., Omanyo D., Musamli R., Kiriga B. (2021). Food security and welfare changes under COVID-19 in Sub-Saharan Africa: impacts and responses in Kenya. Global Food Secur..

[32] Karlan D., Zinman J. (2010). Expanding credit access: using randomized supply decisions to estimate the impacts. Rev. Financ. Stud..

[33] Karlan D., Valdivia M. (2011). Teaching entrepreneurship: impact of business training on microfinance clients and institutions. Rev. Econ. Stat..

[34] Alliance for a Green Revolution in Africa (AGRA) (2013). http://reliefweb.int/sites/reliefweb.int/ﬁles/resources/agraﬁnalaugust20akim.pdf.

[35] Garg K.K., Karlberg L., Barron J., Wani S.P., Rockstrom J. (2011). Assessing the impact of agricultural interventions at the Kothapally watershed, Southern India. Hydrol. Process..

[36] Yeboah E.A., Balcombe K., Asante B.O., Prah S., Aidoo R. (2023). Does participation in innovation platform improve welfare? Insights from smallholder maize farmers in Ghana. African Journal of Science, Technology, Innovation and Development.

[37] Awotide B.A., Alene A.D., Abdoulaye T., Manyong V.M. (2015). Impact of agricultural technology adoption on asset ownership: the case of improved cassava varieties in Nigeria. Food Secur..

[38] Oladejo J.A., Olawuyi S.O., Anjorin T.D. (2011). Analysis of women participation in agricultural production in egbedore local government area of osun state, Nigeria. International Journal of Agricultural Economics and Rural Development.

[39] Nxumalo K.K.S., Oladele O.I. (2013). Factors affecting farmers' participation in agricultural programme in zululand district, kwazulu natal province, South Africa. Journal of Social Science.

[40] Baffoe G., Matsuda H., Nagao M., Akiyama T. (2014). The dynamics of rural credit and its impacts on agricultural productivity: an empirical study in rural Ghana. OIDA Int. J. Sustain. Dev..

[41] Eneyew A. (2012). Determinants of livelihood diversification in pastoral societies of Southern Ethiopia. Journal of Agriculture and Biodiversity Research.

[42] Dzanku F.M. (2015). Household welfare effects of agricultural productivity: a multidimensional perspective from Ghana. J. Dev. Stud..

[43] Kpoor A. (2019). Assets and livelihoods of male- and female- headed households in Ghana. J. Fam. Issues.

[44] Gebrehiwot K.G., Makina D., Woldu T. (2017). The impact of micro-irrigation on households' welfare in the northern part of Ethiopia: an endogenous switching regression approach. Studies in Agricultural Economics.

[45] Tigga A., Mesele K.A., Suneetha P. (2018). The impact of irrigation on poverty alleviation and asset creation in northern Ethiopia. IJCRT.

[46] Abdissa F., Tesema G., Yirga C. (2017). Impact analysis of small scale irrigation schemes on household food security the case of Sibu Sire district in Western Oromia, Ethiopia. Irrigat Drainage Sys Eng.

[47] Anang B.T. (2017).

[48] Ngwako G., Mathenge M., Gido E., Kgosikoma K. (2021). Effect of market participation on household welfare among smallholder goat farmers in Botswana. J. Agribus. Rural Dev.

[49] Chen Z., Huffman W., Rozelle S. (2011). Inverse relationship between productivity and farm size: the case of China. Contemp. Econ. Pol..

[50] Minten B., Barrett C.B. (2008). Agricultural technology, productivity, and poverty in Madagascar. World Dev..

[51] Setsoafia D.E., Ma W., Renwick A. (2022). Effects of sustainable agricultural practices on farm income and food security in northern Ghana. Agricultural and Food Economics.

[52] Orinda M.A. (2013). Analysis of factors inﬂuencing sweet potato value. Analysis of factors inﬂuencing sweet potato value addition amongst smallholders farmers in rachuonyo South district, Kenya. Agriculture Journal.

[53] Gebremariam G., Wünscher T. (2016). Combining sustainable agricultural practices pays off: evidence on welfare effects from Northern Ghana. African Association of Agricultural Economists (AAAE).

[54] Suvedi M., Ghimire R., Kaplowitz M. (2017). Farmers' participation in extension programs and technology adoption in rural Nepal: a logistic regression analysis. J. Agric. Educ. Ext..

[55] Nakano Y., Tsusaka T.W., Aida T., Pede V.O. (2018). Is farmer-to-farmer extension effective? The impact of training on technology adoption and rice farming productivity in Tanzania. World Dev..

[56] Fenger N.A., Bosselmann A.S., Asare R., de Neergaard A. (2017). The impact of certification on the natural and financial capitals of Ghanaian cocoa farmers. Agroecology and Sustainable Food Systems.

[57] Anang B.T., Amikuzuno J. (2015). Factors inﬂuencing pesticide use in smallholder rice production in Northern Ghana. Agric. For. Fish..

